# Letter to the editors: the potential role for prehospital thrombolysis and time-critical stroke transfers in the northern Norway aeromedical retrieval system; In response to: Norum J, Elsbak TM: Air ambulance services in the Arctic: a Norwegian study. *Int J Emerg Med *2011, 4:1

**DOI:** 10.1186/1865-1380-4-45

**Published:** 2011-07-26

**Authors:** Joseph Y Ting

**Affiliations:** 1 Careflight Medical Services, Brisbane 4000, Australia

## Abstract

The role for prehospital thrombolysis for ST-elevation acute myocardial infarction and time-critical stroke transfers in the northern Norway aeromedical retrieval system as well as the aero-evacuation impact of increased Arctic expedition tourism could benefit from further discussion by Norum and Elsbak. Close ECG surveillance for ST elevation and retrieval thrombolysis en route to the accepting hospital could be of benefit for acute coronary syndrome patients in northern Norway who require prolonged aeromedical transfer. For patients who remain within a reasonable time frame for stroke thrombolysis (up to 4.5 h after symptom onset), expedited transfer for neuroimaging to determine eligibility is recommended.

## Letter to the Editors

Norum and Elsbak describe the clinical and transfer characteristics of fixed and rotary wing aeromedical retrievals carried out in northern Norway for the 10 years 1999-2009 [1]. Acute cardiovascular diagnoses were encountered in 76/345 (22%) of the transported cohort [1,2], although it is not stated which, if any, of these patients suffered high-risk acute coronary syndromes (ACS) and/or ST elevation acute myocardial infarction (STEMI). With one in five of the transported cohort potentially having a STEMI or high-risk ACS, a lengthy 3 h 33 min one-way transfer time [1] and attendant delays to accessing primary percutaneous reperfusion, Norum and Elsbak's study could have discussed the role of early recognition of ST elevation and prehospital thrombolysis. Prehospital ECG recognition of STEMI is reliable [3], and prehospital thrombolysis is safe [4] and acutely as clinically beneficial as primary angioplasty if transfer times are anticipated to exceed 120 min from onset of chest pain [5-7], a situation that applies to Norum and Elsbak's study cohort. Furthermore, in high-risk ACS, close ECG surveillance for attainment of lysis criteria followed by timely prehospital thrombolysis could mitigate further ST elevation (and the extent of myocardial injury) during transport [8].

Despite the apparent absence of neurological/stroke patients in their 10-year retrieval registry, Norum and Elsbak's [1] emphasis on urgent aeromedical transfer to identify stroke patients suitable for thrombolysis is to be applauded. Stroke lysis with intravenous alteplase remains beneficial when administered at up to 4.5 h after symptom onset [9,10], a time range that remains relevant within the approximately 3.5 h mean transfer time encountered in the northern Norwegian aeromedical system. That rapidly aging adult populations in advanced economies will give rise to increased stroke burden is borne out by the transport of 69 stroke patients among 504 patients (14%) recently retrieved by a single agency German aeromedical service [11].

Norum and Elsbak [1] speculate that increasing expedition ship tourist traffic to Arctic Norway could lead to an increased need for aeromedical evacuation. However, most expedition ships operating in the Arctic are currently physician-staffed, with non-life or limb-threatening respiratory, gastrointestinal, dermatological, musculoskeletal complaints as well as minor trauma being the most frequently encountered health complaints [12]. Cardiovascular and neurological events, emergency evacuation, need for hospitalization as well as unexpected deaths are rarely encountered despite travelers being older, suggesting effective pre-trip medical screening [12]. At this stage it remains uncertain whether more Arctic tourism will necessarily increase aeromedical workload; this has been my experience as ship's physician in Svalbard in July and August 2009, when there was only one case of shipboard IV rehydration required for non-specific enteritis.

## Abbreviations

ACS: acute coronary syndromes; STEMI: ST elevation acute myocardial infarction.

## Competing interests

The authors declare that they have no competing interests.

## Authors' contributions

JT declares that he fulfils all three criteria for authorship: (1) contribution to design, (2) involved in drafting and critically revising manuscript, and (3) approved the final version of manuscript for publication, if accepted.
